# O.5.1-11 Index for Promoting Sustainable, Healthy and Environment friendly Lifestyles and its association with metabolic syndrome: findings from a Swedish Population-Based Cohort Study

**DOI:** 10.1093/eurpub/ckad133.241

**Published:** 2023-09-11

**Authors:** Nisha Singh, Katarina Bälter, Annika Tillander

**Affiliations:** Mälardalen University, Sweden; Mälardalen University, Sweden; Linköping university, Sweden; nisha.singh@mdu.se

## Abstract

**Purpose:**

The purpose of this study was to investigate the association between sustainable and healthy lifestyle factors and the risk of metabolic syndrome, with a focus on promoting sustainable, healthy and environment friendly behaviours. By creating Index for Promoting Sustainable, Healthy and Environment friendly Lifestyles (IPSHEL), we aimed to assess the combined impact of various lifestyle factors on the development of metabolic syndrome.

**Methods:**

A population-based cohort study was conducted in Sweden, involving a total of 5,364 participants. Lifestyle factors related to physical activity, food habits, tobacco use, alcohol, and sleep were assessed through questionnaires. To quantify the influence of these factors, an Index called IPSHEL was developed. Each lifestyle factor was graded and aggregated into an overall index score for each participant.

**Results:**

The results showed a significant association between IPSHEL and the risk of metabolic syndrome. Individuals with the highest IPSHEL scores had reduced relative risk of developing metabolic syndrome compared to those with the lowest IPSHEL scores. Notable lifestyle factors strongly associated with metabolic syndrome included the intake of fast food and soda, portion sizes of meat and other protein-rich foods, intake of red and processed meat, consumption of cakes, candy, chocolate, ice-cream, chips, total sitting time, snuff use, and the climate impact from diet.

**Conclusions:**

This study highlights the importance of adopting a sustainable and healthy lifestyle in reducing the risk of metabolic syndrome. The IPSHEL proved to be a valuable tool in assessing the combined impact of multiple lifestyle factors. These findings suggest the practical application of the index in public health contexts, serving as a useful tool for surveys, interventions, and evaluations targeting healthy behaviours. By promoting sustainable, healthy and environment friendly lifestyle choices, we can strive towards improved population health and the achievement of global development goals.

